# Plasma Amino-Terminal Propeptide of C-Type Natriuretic Peptide Concentration in Normal-Weight and Obese Children

**DOI:** 10.4274/jcrpe.4543

**Published:** 2017-12-15

**Authors:** Seda Topçu, Bayram Özhan, Afra Alkan, Mesut Akyol, Filiz Şimşek Orhon, Sevgi Başkan, Betül Ulukol, Merih Berberoğlu, Zeynep Şıklar, N. Lale Şatıroğlu Tufan, A. Çevik Tufan

**Affiliations:** 1 Ankara University Faculty of Medicine, Department of Pediatrics, Division of Social Pediatrics, Ankara, Turkey; 2 Pamukkale University Faculty of Medicine, Department of Pediatric Endocrinology, Denizli, Turkey; 3 Ankara Yıldırım Beyazıt University Faculty of Medicine, Department of Biostatistics, Ankara, Turkey; 4 Ankara University Faculty of Medicine, Department of Pediatric Endocrinology, Ankara, Turkey; 5 Ankara University Faculty of Medicine, Department of Forensic Medicine, Forensic Genetics Laboratory, Ankara, Turkey; 6 Ankara University Faculty of Medicine, Department of Pediatric Genetics, Molecular Genetics Laboratory, Ankara, Turkey; 7 Ankara Yıldırım Beyazıt University Faculty of Medicine, Department of Histology and Embryology, Ankara, Turkey

**Keywords:** C-type natriuretic peptide, amino-terminal propeptide of C-type natriuretic peptide, obesity, overweight, growth, biomarker

## Abstract

**Objective:**

In studies on the relationship between amino-terminal propeptide of C-type natriuretic peptide (NT-proCNP) concentration and height velocity in children, CNP has been implicated as an emerging new growth marker during childhood. It has been reported that besides its well-studied role in growth, plasma CNP levels are reduced in overweight and/or obese adolescents, suggesting CNP as a potential biomarker in childhood obesity. The primary goal of this study was to test this hypothesis in a Turkish population.

**Methods:**

Consent was taken from 317 children [ages 0-18 (158 girls, 159 boys)] and their parents. All subjects were physically examined; anthropometric measurements were obtained. Body mass index was calculated. During routine blood work, 1 mL extra blood was taken. Plasma NT-proCNP concentration was measured by enzyme-linked immunosorbent assay.

**Results:**

Results confirmed the previously described relationship between plasma NT-proCNP concentration and growth velocity. Plasma NT-proCNP concentration showed a negative correlation with age, weight, and height in children. Gender was not a factor that alters the age-dependent plasma NT-proCNP concentration until puberty.

**Conclusion:**

Unlike previous reports, plasma NT-proCNP concentration of overweight/obese children was not significantly lower than that of children with normal weight in age groups analyzed in a Turkish population. Thus, it is too early to conclude that CNP is a potential biomarker in childhood obesity. Further studies are necessary to address this question.

**What is already known on this topic?**

Results confirmed the previously described relationship between plasma amino-terminal propeptide of C-type natriuretic peptide (NT-proCNP) concentration and growth velocity. Plasma NT-proCNP concentration showed a negative correlation with age, weight, and height in children. Gender was not a factor that alters the age-dependent plasma NT-proCNP concentration until puberty.

**What this study adds?**

In contrast to what has been suggested before, plasma NT-proCNP concentration of children with overweight/obesity was not significantly lower than that of children with normal weight in age groups analyzed in a Turkish population. Thus, it is too early to conclude that CNP is a potential biomarker in childhood obesity. Further studies are necessary to address this question.

## INTRODUCTION

The amino-terminal propeptide of C-type natriuretic peptide (NT-proCNP) has mainly been implicated as a paracrine/endocrine factor involved in regulation of endochondral growth (1,2,3,4,5,6,7,8,9,10,11,12,13,14,15). The relationship between plasma CNP concentration and height velocity in children has been demonstrated, and CNP has been implicated as an emerging new growth marker during childhood (5,12,13). Besides its well-studied role in growth, recent investigations also relate CNP and the signaling pathway induced by this peptide with obesity (16,17,18,19). These studies have shown that plasma CNP levels are reduced in adolescents with overweight and/or obesity, suggesting CNP as a potential biomarker in childhood obesity.

The identification of CNP was based on its structural similarity to atrial natriuretic peptide (ANP) and brain natriuretic peptide (BNP) (20). Despite their structural similarity, natriuretic peptides are functionally distinct hormones (8). The first two, ANP and BNP, are produced by the atrium and the ventricle, respectively. They act mainly as cardiac hormones (6). CNP, on the other hand, is detected in tissues of a wide variety of systems in the body including the skeletal, central nervous, cardiovascular, urogenital, and immune systems (21). Recent investigations are focusing on the fact that CNP may be used as a potential biomarker related to disease conditions of at least some of these tissues and systems in the body (21). In this context, two recent studies which were performed by the same group analyzed plasma CNP concentration of adolescents with normal weight versus overweight and/or obesity in an Italian population (18,19). They observed lower plasma CNP levels in children with overweight/obesity, suggesting “a defective natriuretic peptide system in these patients” (18).

Childhood obesity is a continuously growing health problem, being considered as a major risk factor for dyslipidemia, hypertension, and damaged glucose metabolism, early onset of endothelial dysfunction, atherosclerosis, and cardiovascular diseases (22,23,24). Based on this knowledge and previous reports (18,19), it was considered important to evaluate CNP as a potential biomarker in early detection of obesity-related disease conditions during child growth. However, the half-life of CNP in the circulation is very short, approximately 2 minutes (21). It has been shown that the proCNP is also secreted from the cell in equal molar concentrations as CNP (25,26). In addition, proCNP is more stable in the circulation, allowing accurate measurement and estimation of CNP concentration (12). It is also known that degradation of proCNP in the circulation starts from the carboxyl terminal of the peptide (12) and N-terminal-directed antibodies against proCNP increase specificity for the propeptide (21). Thus, NT-proCNP is considered as an accurate target for the measurement of circulating CNP concentration and as a potential biomarker in growth and/or human diseases (12,21,27).

The first two studies relating the plasma CNP concentration with obesity have been performed on early adolescents (18,19). Almost all studies relating CNP with growth, on the other hand, have been performed on a variety of age groups of children (5,12,13,28,29). The primary goal of the present study was to analyze plasma NT-proCNP concentration in healthy Turkish normal-weight and overweight/obese children in a broad spectrum of age groups and evaluate the value of NT-proCNP as a potential biomarker in childhood obesity.

Reports within the last decade indicate that weight gain is an important and growing childhood problem also in Turkey (30,31,32,33). Since previous studies have demonstrated the importance of use of population-specific data for the evaluation of age-related changes in growth parameters during childhood (34,35,36), the existence of recently updated Turkish population-specific growth charts was an important advantage for this study (30,31,32,33).

## METHODS

### Subjects

Subjects were children of ages between 0 and 18 years (158 girls and 159 boys) recruited from those attending the Outpatient Clinic of the Pamukkale University Hospital Pediatric Endocrinology Unit in Denizli, Turkey and the Outpatient Clinics of Ankara University Children’s Hospital Social Pediatrics and Pediatric Endocrinology and Adolescent Departments in Ankara, Turkey. The study was approved by the Institutional Clinical Ethics Review Board of Pamukkale University Faculty of Medicine, Denizli, Turkey (decision dated 27.05.2014 and numbered 2014/08, approval number: 1). Written consent was taken from all participants and/or parents/legal guardians of the participating children.

### Establishment of the Age Groups Studied

Since previous studies have correlated plasma CNP/NT-proCNP concentration primarily with height velocity, the age groups in this study were established according to the general knowledge on height velocity changes during childhood (34,35,36,37). In addition, Turkish population-specific age-dependent height velocity changes were also analyzed from the existing growth charts of Turkish children (31,32,33). Age groups established on the basis of these sources were: 1) 0-1 month (newborns), 2) 1-12 months, 3) 1-4 years, 4) 4-10 years, 5) 10-12.5 years, 6) 12.5-14.5 years, and 7) 14.5-18 years. The study model established by these age groups well represented the age-dependent change in height velocity in Turkish children.

All age groups except newborns were further divided into subgroups according to body mass index (BMI) percentiles (38,39,40,41). The statistical percentiles were used to identify overweight (≥95th percentile) up to 2 years of age and obesity (≥95^th^ percentile), overweight (85^th^ to 95^th^ percentile), and normal-weight (5^th^ to 85^th^ percentile) groups in the 2-18 years age range (38,39,40,41). The statistical percentiles of Turkish children published previously were used in all these procedures as the population-specific guidelines (30,31,32). Inclusion criteria of healthy children for this study were as described previously (12,13).

### Study Procedures

All children were seen in the participating outpatient clinics; family and medical histories were obtained. A physical examination was performed in all subjects. Anthropometric measurements including length/height (length by recumbent stadiometer for subjects younger than 2 years old, height by Harpenden stadiometer) and weight (by electronic scale) were obtained (12) in all subjects. BMI (kg/m^2^) was calculated. Percentiles and standard deviation scores were determined according to the Turkish population-specific growth charts (32).

### Analysis of Plasma Amino-Terminal Propeptide of C-Type Natriuretic Peptide Concentration

Venous blood (1 mL) was drawn into tubes containing ethylenediaminetetraacetic acid and processed within two hours. Plasma was isolated by centrifugation of the blood for 10 min at 2000 g. Plasma samples in which hemolysis was observed were excluded from the study. The plasma samples were stored at -80 oC in aliquots until assayed. Each sample was assayed at least twice, and mean value was calculated for each sample. New aliquots were used for each assay. Commercially available “Enzyme Immunoassay for the Quantitative Determination of Human NT-proCNP in Plasma and Serum” (Biomedica Medizinprodukte GmbH & Co KG, Vienna, Austria, Cat. No. BI-20872) was used according to the manufacturers protocol and as reported previously (12).

**Figure 1:**
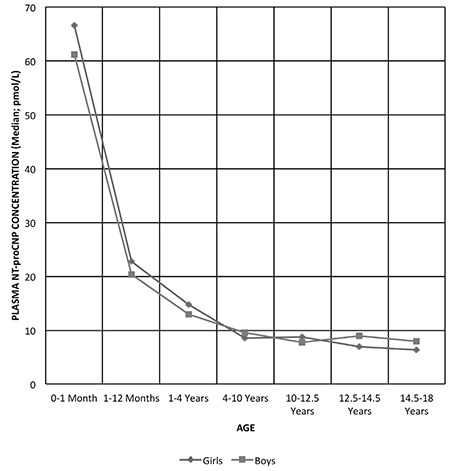
Age-dependent changes in plasma amino-terminal propeptide of C-type natriuretic peptide (NT-proCNP) concentration. Children of normal weight [146 children (76 girls, and 70 boys)] in the 7 different age groups were used to analyze the age-dependent changes in plasma NT-proCNP concentration

The normal-weight group (abbreviated as NW-group for the rest of this report) consisted of 146 children (76 girls and 70 boys) in the established 7 different groups. The data on this group were used to analyze the age-dependent changes in plasma NT-proCNP concentration (Figure 1), and comparison of plasma NT-proCNP concentration based on gender in each age group (Table 1). Plasma NT-proCNP concentrations in the NW-group [136 children (71 girls and 65 boys)] were compared with those of children with overweight/obesity [abbreviated as the OW/O-group for the rest of this report; 171 children (82 girls and 89 boys)] in all groups except for the newborns [10 children (5 girls and 5 boys)] (Table 2).

### Statistical Analysis

Shapiro-Wilk test was used to analyze the distribution pattern of the continuous variables in this study. No assumptions of normal distribution of the data were made. Comparison of NT-proCNP concentration within each age group of NW-group based on gender was performed by either Mann-Whitney U test or t-test, based on the distribution of NT-proCNP concentration in age groups. For the correlations of NT-proCNP concentration of NW-group with age, weight, and height, Spearman rho correlation coefficients were calculated. Comparison of NT-proCNP concentration within each age group based on BMI, i.e., NW-group vs. OW/O-group, was performed by Mann-Whitney U test. All these analyses were carried out using IBM SPSS Statistics 21.0 software (IBM Corp. Released 2012. IBM SPSS Statistics for Windows, Version 21.0. Armonk, NY: IBM Corp.). Statistical significance level was taken as p<0.05.

## RESULTS

### Comparison of Amino-Terminal Propeptide of C-Type Natriuretic Peptide Concentration within Each Age Group of NW-Group Based on Gender

Analysis of plasma NT-proCNP concentrations in the different age groups of healthy girls and boys in the NW-group showed that gender was not a factor that alters the age-dependent plasma NT-proCNP concentration until puberty in this group (Figure 1; Table 1). In subjects older than 12.5 years, plasma NT-proCNP concentrations were higher in boys than in girls, and in the 14.5-18-year-old group, this difference was statistically significant (p<0.05).

### Correlations within the NW-Group of Children

Plasma NT-proCNP concentration was negatively correlated with age (n=146; r=-0.878; p<0.001), weight (n=146; r=-0.863; p<0.001), and height (n=146; r=-0.866; p<0.001) in the NW-group.

### Comparison of Amino-Terminal Propeptide of C-Type Natriuretic Peptide Concentration within Each Age Group Based on Body Mass Index

Plasma NT-proCNP concentrations in the NW-group and the OW/O-group were compared in each age group. The results revealed that the plasma NT-proCNP concentrations of girls and boys in the OW/O-group did not differ from that of their peers in the NW-group at any age group studied (Table 2).

Since two previous studies analyzed plasma CNP concentration of adolescents with overweight/obesity at a very narrow age interval, i.e., 11.8±0.4 years for the first study (18) and 12.8±2.4 years for the second study (19), a similar analysis was also performed in this study in a group of 109 children [31 children with normal weight (19 girls and 12 boys) and 78 children with overweight/obesity (34 girls and 44 boys)] at age 12.64±1.58 years. The results revealed once again that mean plasma NT-proCNP concentration in children of normal weight (8.22±1.85 pmol/L) was comparable to that of children with overweight/obesity (8.76±2.72 pmol/L).

## DISCUSSION

The primary goal of the present study was to analyze and compare, in a Turkish population of different age groups, plasma NT-proCNP concentrations in a NW-group and an OW/O-group and to evaluate NT-proCNP as a biomarker in childhood obesity.

To test the reliability of plasma NT-proCNP concentrations obtained in this study, a confirmatory first experiment was designed, during which the plasma NT-proCNP concentration of healthy children of normal weight was analyzed through an age-dependent group model (Figure 1, and Table 1). The goal was to demonstrate, in a Turkish population, the previously published relationship between plasma CNP concentration and height velocity during child growth (5,12,13,28,29,42). In the second experiment, on the other hand, according to the above-mentioned primary goal of this study, an age- and gender-matched comparison was made between the NW-group and the OW/O-group in terms of plasma NT-proCNP concentrations.

The technique used to analyze plasma CNP and/or NT-proCNP concentration has been one of the most variable part of the studies published previously in this field. Two generally accepted applications include radioimmunoassay (RIA) (5,12,13) and enzyme-linked immunosorbent assay (ELISA) (2). A previously evaluated (12) commercially available ELISA kit was used in this present study for the analysis of plasma NT-proCNP concentration. It was reported that the correlation between the RIA for NT-proCNP and this commercially available ELISA kit for NT-proCNP was significant (r=0.748, p<0.0005) (12). However, “the commercial ELISA” revealed values that were, on the average, 21% of the RIA values (range, 11-52%) (12). When evaluated in this context, plasma NT-proCNP concentrations obtained in girls and boys in the NW-group at different age groups (Figure 1; Table 1) were comparable to data reported by Olney et al (12). In addition, as reported previously (5,12,13,28,29,42), our results also showed that in children, plasma NT-proCNP concentration was negatively correlated with age, weight, and height. Gender was not a factor that alters the age-dependent plasma NT-proCNP concentration until puberty in the NW-group. After 12.5 years of age, plasma NT-proCNP concentration was higher in boys than in girls. All these results were in agreement with the literature (5,12,13,28,29,42), a finding supporting the appropriate design and also the reliability of the measurement of plasma NT-proCNP concentrations in this study.

To evaluate NT-proCNP as a potential biomarker in early detection of obesity-related disease conditions during child growth, a sex- and age-matched comparison was performed between the plasma NT-proCNP concentration of the OW/O-group and that of the NW-group. Plasma NT-proCNP concentration of girls, boys, and of children overall (girls + boys) in the OW/O-group did not differ from that of NW-group at any age group studied.

Since two previous studies, which suggested a lower plasma CNP concentration in adolescents with overweight/obesity, analyzed plasma CNP concentration at a very narrow age interval, i.e., 11.8±0.4 years for the first study (18) and 12.8±2.4 years for the second study (19), a similar analysis was also performed in this study in a group of 109 children aged 12.64±1.58 years. This age interval was important in terms of reflecting the period at which growth velocity peaks during puberty both in girls and boys. Results revealed once again that mean plasma NT-proCNP concentration in the NW-group (8.22±1.85 pmol/L) was comparable to that of the OW/O-group (8.76±2.72 pmol/L).

Based on these results, it was concluded that plasma NT-proCNP concentration may be an important growth marker during childhood as suggested in the literature (5,12,13,28,29,29). However, unlike previous statements (18,19), in our subjects, plasma NT-proCNP concentration of the NW-group and the OW/O-group did not differ from one another at any age group studied, including adolescents.

The literature is very limited in terms of CNP and its relation to obesity. Other than two studies that suggested lower plasma CNP levels in adolescents with overweight/obesity (18,19), there are some studies which suggest melanocortin receptors as targets in the treatment of obesity (16), and CNP as a melanocortin receptor analog in mice (17). Yamada-Goto et al (17) reported that intracerebroventricular administration of CNP suppresses food intake via activation of the melanocortin system in mice. There are also some studies that investigated the relation between CNP and hypercholesterolemia (43), for which obesity is considered as a major risk factor. However, it has been shown that systemic BNP and CNP levels are not altered in patients affected by hypercholesterolemia (43).

### Study Limitations

In terms of study limitations, this study was performed only on Turkish children. Additionally, a previously evaluated (12) commercially available ELISA kit was used in this present study for the analysis of plasma NT-proCNP concentration. It has been reported that “the commercial ELISA” revealed values that were, on the average, 21% of the RIA values (range, 11-52%) (12).

On the other hand, the technique used to analyze plasma CNP and/or NT-proCNP concentration has been one of the most variable part of the studies published previously in this field. Two generally accepted applications include RIA (5,12,13) and ELISA (12). It was reported that the correlation between the RIA for NT-proCNP and the commercially available ELISA kit for NT-proCNP was significant (r=0.748, p<0.0005) (12).

Our findings show that unlike previous reports, plasma NT-proCNP concentration of overweight/obese children was not significantly lower than that of children with normal weight in age groups analyzed in a Turkish population. However, the conclusion established in our study should be confirmed in subsequent studies.

## CONCLUSION

At this stage, it is clear that CNP signaling may somehow be related to obesity and/or its treatment strategies. However, it is too early to conclude that it is a potential biomarker in the early detection of obesity and/or obesity-related disease conditions during child growth. Future studies are necessary to address this question.

## Figures and Tables

**Table 1:**
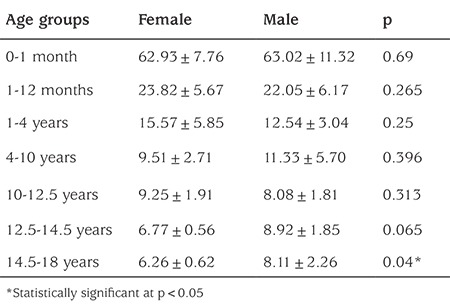
Amino-terminal propeptide of C-type natriuretic peptide concentration in children of normal weight based on gender in each age group

**Table 2:**
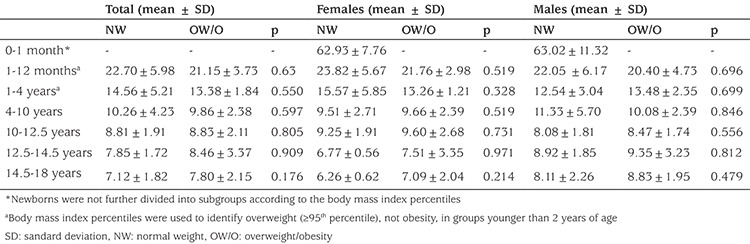
Comparison of amino-terminal propeptide of C-type natriuretic peptide concentration by body weight classification within each age group of children
